# Mosaic ratio quantification of isochromosome 12p in Pallister–Killian syndrome using droplet digital PCR

**DOI:** 10.1002/mgg3.200

**Published:** 2016-01-20

**Authors:** Katsunori Fujiki, Katsuhiko Shirahige, Maninder Kaur, Matthew A. Deardorff, Laura K. Conlin, Ian D. Krantz, Kosuke Izumi

**Affiliations:** ^1^ Research Center for Epigenetic Disease, Institute for Molecular and Cellular BiosciencesThe University of TokyoTokyoJapan; ^2^ Division of Human GeneticsThe Children's Hospital of PhiladelphiaPhiladelphiaPennsylvania; ^3^ The Perelman School of Medicine at The University of PennsylvaniaPhiladelphiaPennsylvania; ^4^ Department of Pathology and Laboratory MedicineThe Children's Hospital of PhiladelphiaPhiladelphiaPennsylvania19104

**Keywords:** Copy number variation, isochromosome 12p, mosaicism

## Abstract

**Background:**

Pallister–Killian syndrome (PKS) is a prototypic mosaic aneuploidy syndrome caused by mosaic supernumerary marker isochromosome 12p. Cells possessing the isochromosome 12p rapidly diminish after birth in the peripheral blood, often necessitating a skin biopsy for diagnosis. Therefore, a genomic testing that is capable of detecting low percent mosaic isochromosome 12p is preferred for the diagnosis of PKS.

**Methods:**

The utility of the droplet digital PCR system in quantifying the mosaic ratio of isochromosome 12p in PKS was evaluated.

**Results:**

Droplet digital PCR was able to precisely quantify isochromosome 12p mosaic ratio, and copy number measured by droplet digital PCR was correlated well with that of fluorescence in situ hybridization analysis.

**Conclusion:**

Droplet digital PCR should be considered as an effective tool for both clinical and research analytics to precisely quantify mosaic genomic copy number alterations or mosaic mutations.

## Introduction

Pallister–Killian syndrome (PKS) (MIM# 601803) is a multisystem developmental disorder characterized by facial dysmorphism, intellectual disability, congenital heart defects, and numerous other birth defects (Izumi and Krantz 2014). PKS is typically caused by the presence of a supernumerary marker isochromosome 12p (iso12p). Based on single nucleotide polymorphism (SNP) genotyping information, the iso12p is believed to be present at the time of the fertilization, and this marker chromosome is gradually lost in some cell types during embryogenesis. Cells possessing the iso12p rapidly diminish after birth in the peripheral blood, often necessitating a skin biopsy for diagnosis (Conlin et al. 2012). We have previously demonstrated the utility of genome‐wide SNP arrays in making a diagnosis of PKS, given its ability to detect lower levels of mosaicism than standard karyotyping (Conlin et al. 2012). In this article, we report the utility of the droplet digital PCR (ddPCR) system in quantifying the mosaic ratio of iso12p in PKS.

**Figure 1:**
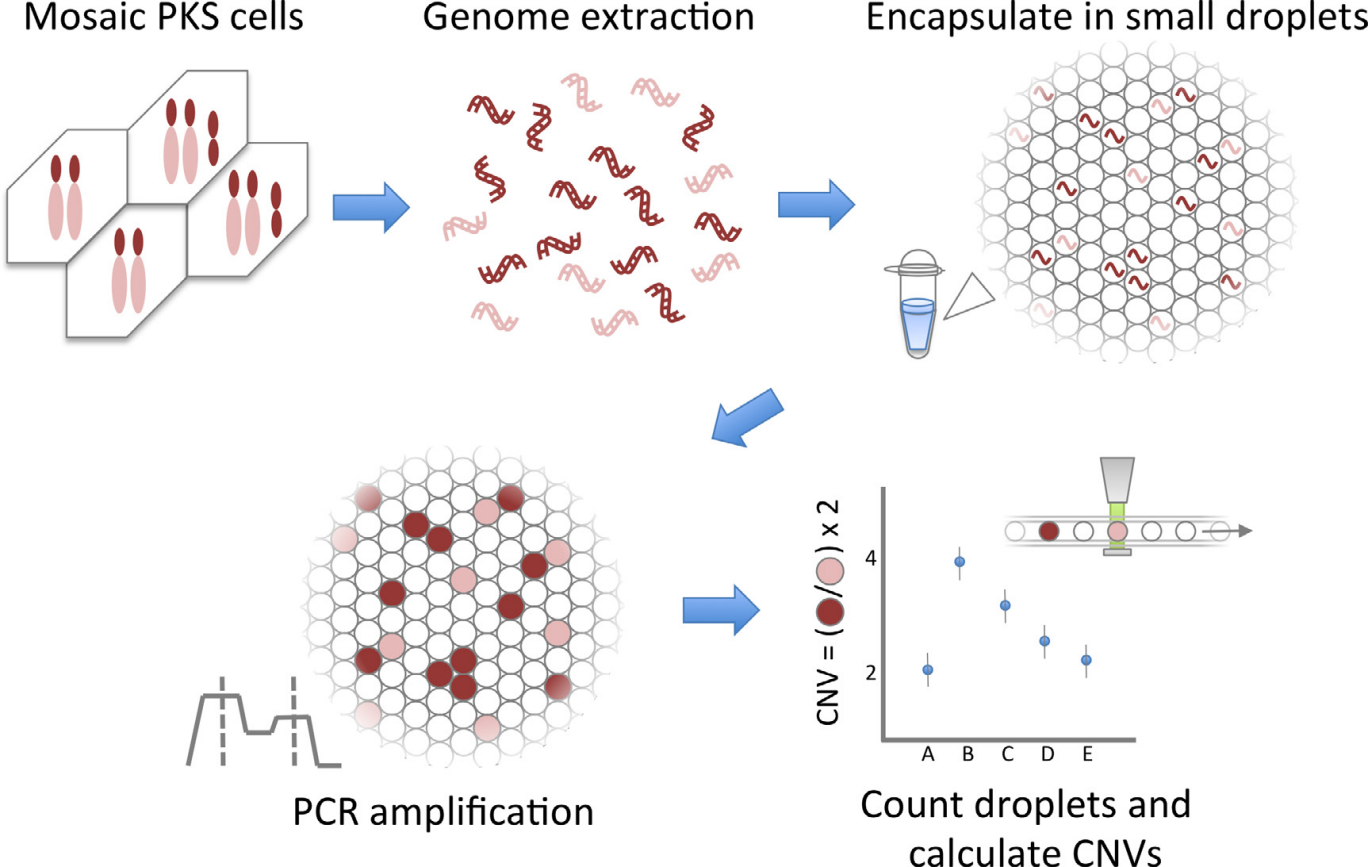
Schema of Pallister–Killian syndrome PKS) copy number evaluation workflow.

ddPCR utilizes a water–oil emulsion droplet technology that compartmentalizes the PCR reaction solution into 15,000–20,000 droplets (Pinheiro et al. 2012). After the droplets are generated, PCR amplification occurs in each droplet using fluorescent dyes such as FAM or VIC and PCR primers. The ddPCR system then quantifies the number of signal positive and negative droplets that allows for the absolute amount of the target genomic region of interest to be counted precisely (Fig. 1). The ability of ddPCR to provide an absolute quantitation of a defined sample makes it a potentially useful technology to detect low levels of the iso12p and quantify the mosaic ratio as well as serve as a rapid diagnostic tool for PKS and other mosaic diagnoses.

## Materials and Methods

### Ethical compliance

All of the PKS probands were enrolled under an institutional review board approved protocol at The Children's Hospital of Philadelphia.

DNA samples from the PKS probands and control individuals were extracted from their skin fibroblast cell lines and lymphoblastoid cell lines using NucleoSpin^®^ tissue kit (Macherey‐Nagel, Düren, Germany). Approximately, 50 ng of genomic DNA was used for the ddPCR reaction. FAM fluorescent tagged TaqMan^®^ copy number assay probes detecting the *ING4* gene (Hs02490252_cn) (Life Technologies, Carlsbad, CA) was used to quantify the copy number of 12p. *ING4* resides within the previously defined PKS critical region of 12p13.31 (Izumi et al. 2014). 12p copy numbers were also evaluated with two other probes targeting *ETNK1* gene at 12p12.1 (Hs01860263_cn) and *DNM1L* gene at 12p11.21 (Hs00527169_cn). There were no copy number variations registered in the Database of Genomic Variants Website (DGV) (http://dgv.tcag.ca/dgv/app/home) spanning these *ETNK1* and *DNM1L* loci, however, two individuals with *ING4* copy number alterations (one partial duplication and one whole gene deletion) were listed in the DGV. In order to evaluate 12q copy number, a FAM fluorescent tagged TaqMan^®^ copy number assay probe detecting *TRPV4* gene locating at 12q24.11 (Hs02348033_cn) was used. VIC fluorescent tagged TaqMan^®^ Copy Number Reference Assay RNase P was used as the internal control (Life Technologies). The ddPCR system was operated according to the manufacturer's instruction. Briefly, the PCR reaction solution was compartmentalized by a droplet generator, and then, PCR amplification performed. The PCR conditions were as follows: 95°C 10 min, followed by 40 cycles of 94°C 30 sec and 57°C or 58°C 1 min, then 98°C for 10 min. After the PCR amplification was completed, a droplet reader counted the number of droplets that were positive and negative for FAM and VIC fluorophore. ddPCR was run in triplicates. The mosaic ratio was calculated based on the total positive signal counts of *ING4, ETNK1*, or *DNM1L* normalized against the RNase P positive signal counts.

For copy number analysis of buccal swab samples, FAM fluorescent tagged TaqMan^®^ copy number assay probe detecting the *ATN1* gene (Hs02339066_cn) was used. Genomic DNA was extracted from the buccal swab sample using Qiagen Puregene Buccal Cell Core Kit A (Qiagen, Valencia, CA).

## Results

**Figure 2:**
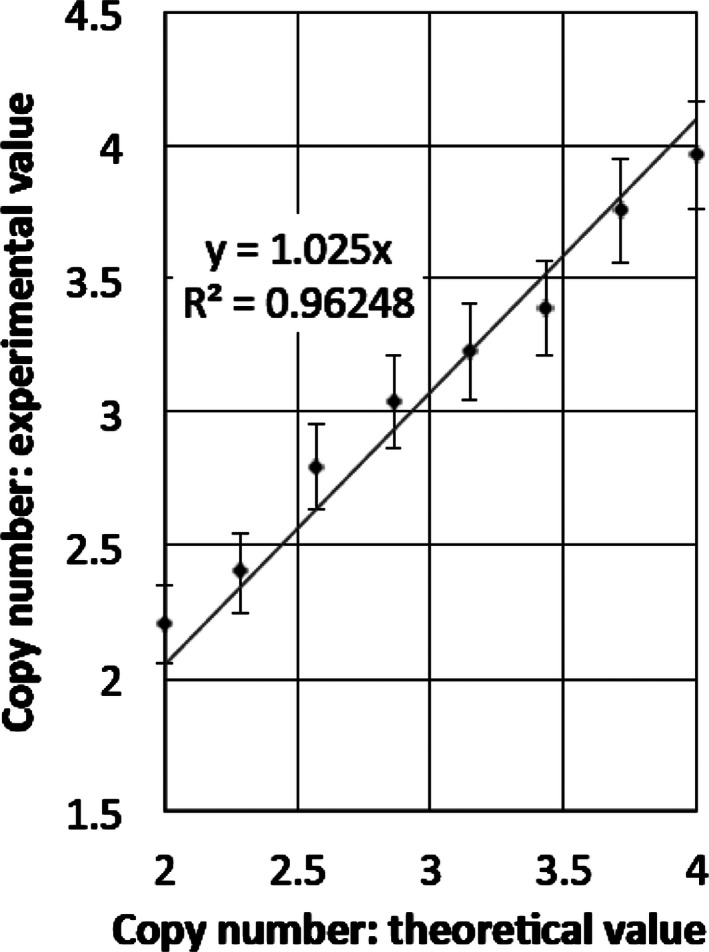
Correlation between ddPCR results and theoretical copy number values. *X*‐axis represents theoretical copy number based on the mix ratio between tetrasomic DNA sample and control disomic DNA sample. *Y*‐axis represents the ddPCR results. Error bars indicate ±2 SD.

We generated a standard curve making use of the genomic DNA obtained from an isolated pure iso12p tetrasomic PKS cell clone, and control genomic DNA. By mixing 12p tetrasomic DNA and control DNA at various ratios, we created DNA samples with defined mosaic levels of iso12p. There was a strong correlation between the ddPCR determined copy number and the theoretical copy number (Fig. 2). In order to evaluate interassay variations of 12p copy number measured by ddPCR, 12p copy number was evaluated using nine additional control genomic DNA samples (seven skin fibroblast samples and two lymphoblastoid cell line samples) and *ING4* probe (Fig. S1). Average 12p copy number of control samples was 2.008 (ranging from 1.95–2.04) and its standard deviation was 0.0326.

**Figure 3:**
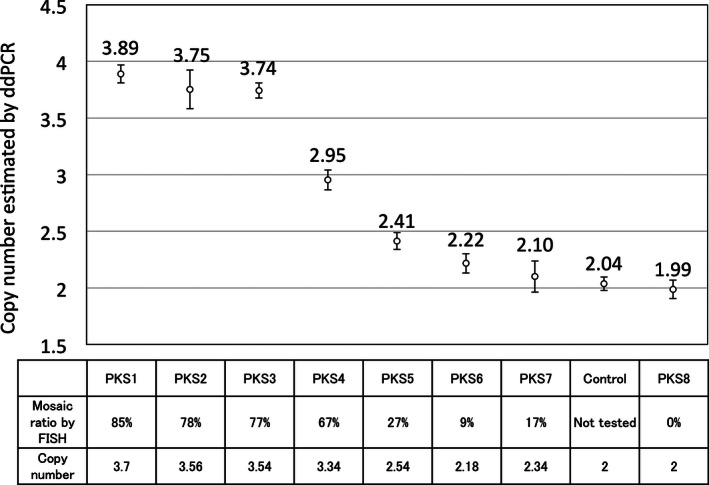
Comparison between ddPCR and fluorescence in situ hybridization (FISH) mosaic ration estimation. *Y*‐axis represents the ddPCR results. Error bars indicate ±2 SD.

In order to evaluate the accuracy of ddPCR mosaic ratio quantification, we compared the mosaic ratio obtained by ddPCR and fluorescence in situ hybridization (FISH) analysis using eight PKS samples and one control sample. Chromosome preparations from cultured fibroblasts were analyzed by FISH using a chromosome 12p‐specific DNA probe and control probe (11q‐specific probe or 19q‐specific probe). FISH was performed according to the standard protocol (DeScipio et al. 2005). For each PKS cell line, 100 cells were manually counted, and the mosaic ratio was obtained. The comparison of mosaic ratio between FISH and ddPCR revealed positive correlation between the two assays (Fig. 3).

**Figure 4:**
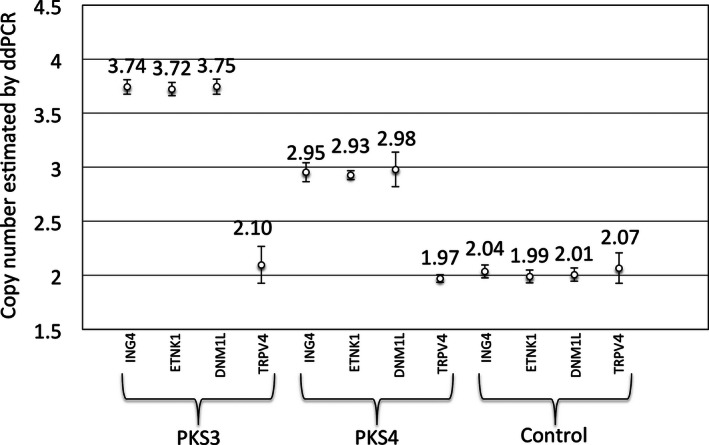
Comparison among three 12p probes and one 12q probe. *Y*‐axis represents the ddPCR results. Error bars indicate ±2 SD. Results of PKS3, PKS4 and control samples.

For the evaluation of the copy number of other regions of 12p, utilizing probes targeting the *ETNK1* gene at 12p12.1 and the *DNM1L* gene at 12p11.21, the ddPCR results were compared to that of *ING4* gene at 12p13.31 using two PKS samples as well as the control sample. The estimated mosaic ratio was highly concordant between these three probes, consistent with the fact that PKS is caused by tetrasomy of the entire arm of 12p (Fig. 4). The copy number assay was also performed with 12q probe (*TRPV4*), and the copy number of 12q was normal in all three samples.

Lastly, since FISH analysis using buccal swab sample is often used for clinical diagnosis of PKS, we performed ddPCR analysis with buccal swab DNA samples. 12p copy number analysis of two control individuals revealed normal copy number, demonstrating that ddPCR 12p copy number analysis can be performed using genomic DNA extracted from the buccal swab samples (Fig. S2).

## Discussion

Here, we demonstrate the utility of ddPCR in quantifying the mosaic ratio of the iso12p marker chromosome in PKS. PKS is a prototypic mosaic aneuploidy syndrome and it is anticipated that these results can be extrapolated to other mosaic aneuploidy diagnoses. Historically, to make a diagnosis of PKS, karyotype G‐band analysis or FISH analysis were used, and more recently, these techniques have been increasingly replaced by CGH or SNP arrays. While these techniques have been shown to be effective diagnostic tools and are able to identify lower level mosaics than previous methodologies, they are limited by their technical complexity, cost, and turnaround time. The rapid turnaround time of ddPCR is very advantageous in a clinical setting. The total time required for ddPCR is about an hour for DNA extraction and 3–4 h for the ddPCR reaction and analysis, therefore, 12p copy number analysis can be completed within several hours. Since the clinical phenotype of PKS is highly conserved and can be suspected on a clinical ground, this type of targeted rapid diagnostic method is valuable. Furthermore, since the amount of DNA sample required is small (only 50 ng for each reaction), ddPCR requires a very small amount of clinical sample and does not require live dividing cells.

Analysis of the 12p probes generally yielded comparable results among the three probes tested in our analysis. However, there are many copy number polymorphisms reported in 12p region in the DGV Website, and if the 12p probes span such polymorphic regions, the precise copy number estimates of ddPCR cannot be achieved. More importantly, not only mosaic isochromosome 12p, but also 12p microduplication can cause PKS (Izumi et al. 2014). Given the possibility of such copy number polymorphisms and 12p microduplication, we recommend using multiple probes for the diagnosis and quantifying PKS mosaicism.

In our evaluation, we felt the lowest level of mosaicism we can confidentially diagnose using ddPCR would be around 5–10%, which is comparable to that of SNP array (Conlin et al. 2010). The SNP array platform, the Illumina Quad610, we previously used for PKS mosaic quantification, had 8849 SNP probes on chromosome 12p (Conlin et al. 2010). The number of the droplet generated by the ddPCR system is around 15,000, therefore, similar level of mosaic detection capability between SNP array and ddPCR can be anticipated.

Since a similar degree of result variation was seen among technical replicates of all three 12p probes tested in this study, this detection limit of 5% is unlikely to be due to probe sequence/specificity, but rather we suspect this is the technical limit of the ddPCR system to detect mosaic copy number variation in PKS. Theoretically, ddPCR should be able to detect a much lower percent mosaicism, given that the ddPCR system counts up to 15,000 droplets. In the analyses of mosaic oncogenic mutation detection, it was reported that ddPCR system is able to detect lower than a few percentage mosaicism (Reid et al. 2015). In these cases, the oncogenic mutation is seen only in the cancer cells, and is not present in the normal cells. However, for PKS (and other constitutional mosaic aneuploidy diagnoses), the FAM‐targeted genomic sequence, normal 12p sequence in the case of PKS, is not only present in iso12p, but also in the structurally normal chromosome 12p.

Previously, utilization of the ddPCR system in defining the chromosomal breakpoints of 22q11.2 deletions syndrome was reported (Hwang et al. 2014). Application of ddPCR is not only limited to copy number alteration, as demonstrated by the ability of detecting rare oncogenic mutation in cancer, and ddPCR represents an useful technology for quantifying mosaic ratio in other mosaic genetic syndromes. Recently, detection of mosaic *MTOR* mutation using ddPCR was reported in focal cortical dysplasia type IIb (Nakashima et al. 2015). With the recent identification of many mosaic genetic disorders (e.g., Proteus syndrome) (Biesecker and Spinner 2013), ddPCR represents a potentially powerful technique to diagnose and study these conditions in a cost‐effective and rapid manner.

In summary, our results demonstrate the utility of ddPCR for mosaic ratio quantification of PKS. ddPCR represents an accurate and rapid method to detect mosaic chromosomal abnormalities. Therefore, ddPCR should be considered as an effective tool for both clinical and research analytics to precisely quantify mosaic genomic copy number alterations or mosaic mutations.

## Conflict of Interest

KI has received speaker's honorarium from Bio‐Rad Laboratories. All other authors have no conflicts of interest to declare.

## Supporting information


**Figure S1.** 12p copy number analysis of control individuals. *Y*‐axis represents the ddPCR results. Error bars indicate ±2 SD.

**Figure S2.** 12p copy number analysis with buccal swab DNA samples from control individuals. *Y*‐axis represents the ddPCR results. Error bars indicate ±2 SD.
