# Supplementary figures and images for: Mosaic ratio quantification of isochromosome 12p in Pallister–Killian syndrome using droplet digital PCR
Source: Mol Genet Genomic Med. 2016 Jan 20;4(3):257–61. doi: 10.1002/mgg3.200 (PMC4867559; doi:10.1002/mgg3.200)

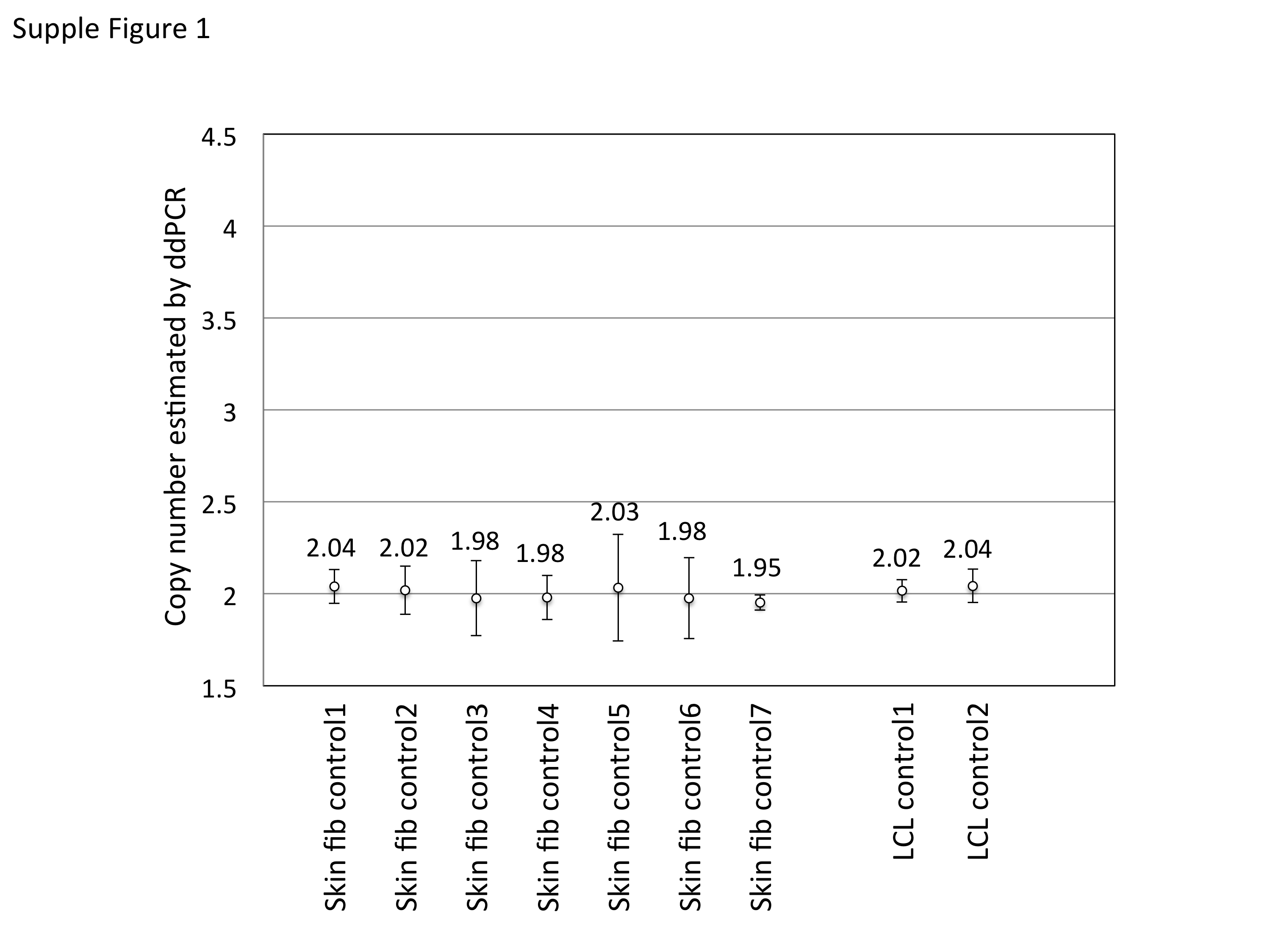

Supplement: Supplementary file 1 — Figure S1. 12p copy number analysis of control individuals. Y‐axis represents the ddPCR results. Error bars indicate ±2 SD. [file MGG3-4-257-s001.tiff]

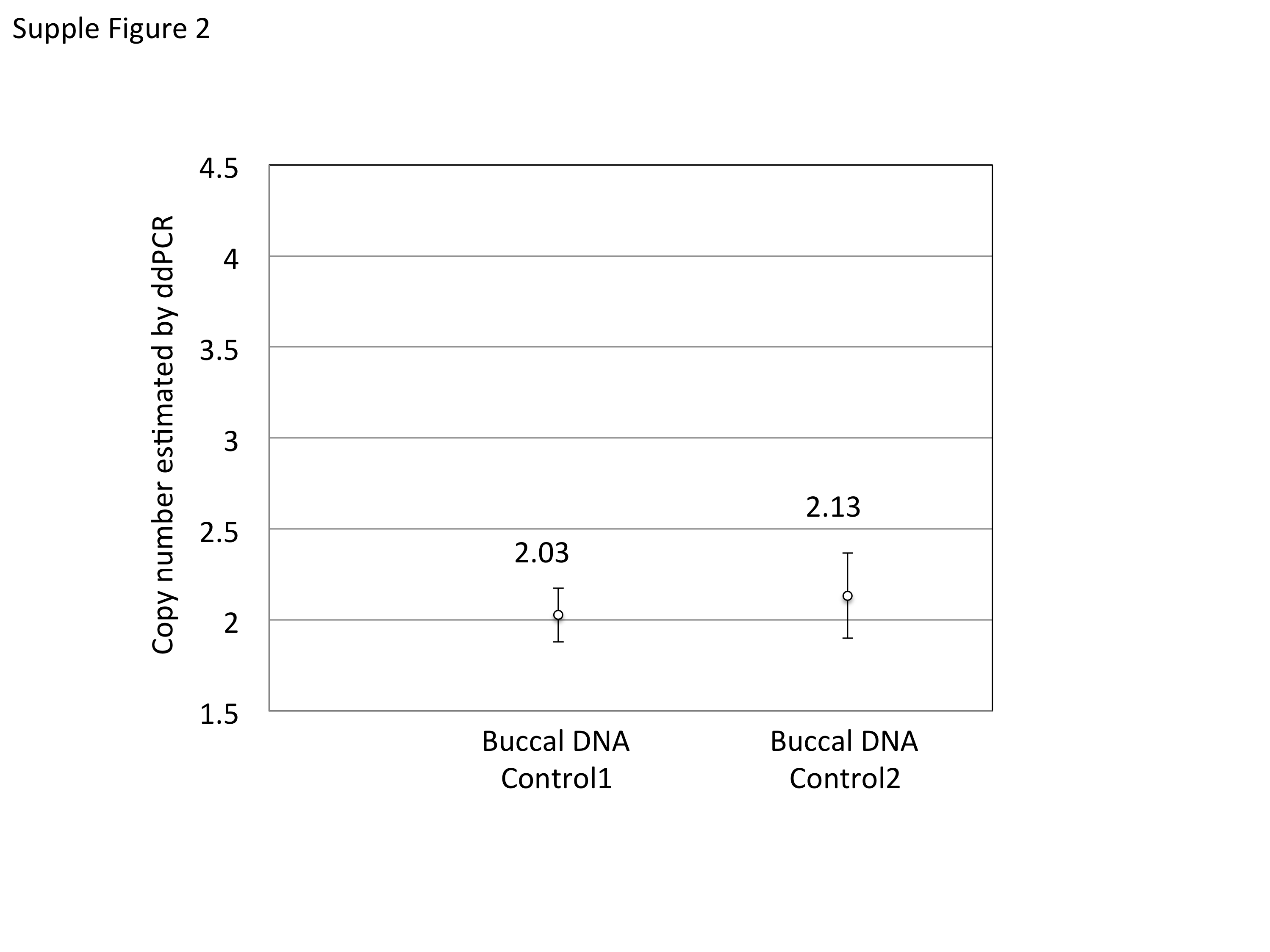

Supplement: Supplementary file 2 — Figure S2. 12p copy number analysis with buccal swab DNA samples from control individuals. Y‐axis represents the ddPCR results. Error bars indicate ±2 SD. [file MGG3-4-257-s002.tiff]
